# Mitochondrial genomes of the stoneflies *Mesonemourametafiligera* and *Mesonemouratritaenia* (Plecoptera, Nemouridae), with a phylogenetic analysis of Nemouroidea

**DOI:** 10.3897/zookeys.835.32470

**Published:** 2019-04-04

**Authors:** Jin-Jun Cao, Ying Wang, Yao-Rui Huang, Wei-Hai Li

**Affiliations:** 1 Department of Plant Protection, Henan Institute of Science and Technology, Xinxiang, Henan 453003, China Henan Institute of Science and Technology Xinxiang China; 2 Postdoctoral Research Base, Henan Institute of Science and Technology, Xinxiang, Henan 453003, China Henan Institute of Science and Technology Xinxiang China

**Keywords:** Amphinemurinae, comparative mitochondrial genomics, Nemouridae, phylogenetics, Plecoptera

## Abstract

In this study, two new mitochondrial genomes (mitogenomes) of *Mesonemourametafiligera* and *Mesonemouratritaenia* from the family Nemouridae (Insecta: Plecoptera) were sequenced. The *Mesonemourametafiligera* mitogenome was a 15,739 bp circular DNA molecule, which was smaller than that of *M.tritaenia* (15,778 bp) due to differences in the size of the A+T-rich region. Results show that gene content, gene arrangement, base composition, and codon usage were highly conserved in two species. Ka/Ks ratios analyses of protein-coding genes revealed that the highest and lowest rates were found in ND6 and COI and that all these genes were evolving under purifying selection. All tRNA genes in nemourid mitogenomes had a typical cloverleaf secondary structure, except for tRNA^Ser(AGN)^ which appeared to lack the dihydrouridine arm. The multiple alignments of nemourid lrRNA and srRNA genes showed that sequences of three species were highly conserved. All the A+T-rich region included tandem repeats regions and stem-loop structures. The phylogenetic analyses using Bayesian inference (BI) and maximum likelihood methods (ML) generated identical results. Amphinemurinae and Nemourinae were sister-groups and the family Nemouridae was placed as sister to Capniidae and Taeniopterygidae.

## Introduction

The mitochondrial genome (mitogenome) of insects is typically a small double-stranded circular molecule of 14–20 kb in size. It contains13 protein-encoding (PCGs), 22 transfer RNA (tRNAs), two ribosomal RNA (rRNAs) genes, and a putative control region (in arthropods, also known as A+T-rich region) where the necessary regulatory sequences for transcription and duplication of the DNA occur (Boore 1999). Because of their simple genomic organization, fast rate of nucleotide substitution, and low levels of sequence recombination, mitogenomes are used as good models in comparative and evolutionary genomics, population genetics, and phylogenetic studies at various taxonomic levels (Lin and Danforth 2004; Gissi et al. 2008; Cameron 2014; Song et al. 2016; Li et al. 2017).

The Plecoptera (stoneflies) comprises an ancient group of insects including about 3,900 described species worldwide (Dewalt et al. 2018). The family Nemouridae, belongs to the superfamily Nemouroidea within the family group Euholognatha in suborder Arctoperlaria (Dewalt et al. 2018). Nemouridae is one of the largest families of Plecoptera with approximately 400 species (Baumann 1975; Dewalt et al. 2018). Baumann (1975) divided the family Nemouridae into two subfamilies, the Amphinemurinae and the Nemourinae, and erected two oriental genera, *Mesonemoura* and *Indonemoura* in the latter subfamily, which also includes the genera *Amphinemura*, *Malenka*, and *Protonemura*. The Amphinemurinae are well distributed in the Palearctic, Nearctic, and Oriental regions. They occur in a wide variety of streams but are probably most diverse in smaller creeks and spring runs (Dewalt et al. 2018). Currently, the position of Nemouridae in Plecoptera has been resolved based on morphology (Illies 1965; Zwick 2000), but several different suggestions are proposed based on molecular data (Thomas et al. 2000; Terry and Whiting 2003; Chen and Du 2018; Wang et al. 2019), and the relationship within the Nemouroidea still lacks precise phylogeny. Up to now, only one nemourid mitogenome (*Nemouranankinensis* Wu, 1926) has been sequenced (Chen and Du 2017a) and those conflicting opinions were mainly generated by the limited mitogenomic data. Therefore, more molecular data is required to reconstruct precise phylogeny (Misof et al. 2014; Wu et al. 2014).

To date, 27 mitogenomes of stoneflies have been sequenced (Wang et al. 2019), but those of the subfamily Amphinemurinae are still not reported, and the phylogeny of Nemouridae is still controversial due to lack of plenty molecular data. To facilitate the resolution of this problem, we report the complete mitogenomes of *M.tritaenia* Li & Yang, 2007 and *M.metafiligera* Aubert, 1967, analyze and compare their mitogenomic organizations, nucleotide compositions, codon usages, RNA secondary structures, Ka/Ks ratios of 13 PCGs, and novel features of the A+T-rich regions. Finally, we also reconstructed the phylogenetic tree of the superfamily Nemouroidea based on the concatenated nucleotide sequences of four datasets (PCGs, PCGR, PCG12 and PCG12R) by using Bayesian inference (BI) and Maximum likelihood (ML) methods, thus our result increases the understanding of stonefly phylogeny.

## Materials and methods

### Specimens and DNA extraction

Adult specimens of *M.tritaenia* and *M.metafiligera* were collected from Baiyun Mountain (Luoyang, Henan Province, China) in July 2015 and from Bolonggong (Tibet, China) in August 2015, respectively. We preserved specimens in 95% ethanol in the field and stored them at – 20 °C until tissues were used for DNA extraction. Voucher specimens of *M.tritaenia* (No. Vhem–0008) and *M.metafiligera* (No. Vhem–0061) were deposited in Entomological Museum of Henan institute of Science and Technology (HIST), Henan Province, China. Specimens were identified by Wei-Hai Li (HIST). We extracted total genomic DNA from thoracic muscle tissue using QIAamp DNA Blood Mini Kit (Qiagen, Duesseldorf, Germany) according to the manufacturer’s protocols.

### Genome sequencing, assembly, and annotation

The mitogenomes were amplified and sequenced as described in previous studies (Song et al. 2016; Li et al. 2017; Wang et al. 2017a, 2017b; Wang et al. 2018a, 2018b). From the genomic DNA, an Illumina TruSeq library with an insert size of 480 bp was generated. We sequenced our library on a single lane of Illumina Hiseq 2500 with 500 cycles of paired-end sequencing (250 bp reads). High-quality reads were generated using Trimmomatic v0.30 (Lohse et al. 2012). Then, IDBA-UD (Peng et al. 2012) was used for de novo assembling with these reads. A similarity threshold of 98% and minimum and maximum k values of 80 and 240 bp, respectively, were used to build assemblies. COI and srRNA fragments were amplified as bait sequences using PCR (Li et al. 2012; Wang et al. 2014) and previously designed primers (Simon et al. 2006) were used to determine the mt genome components. Raw sequences from the mitochondrial genome of each species were assembled into contigs with BioEdit 7.0.5.3 (Hall 1999). We identified protein-coding genes and two ribosomal RNA genes using BLAST searches in NCBI and by alignment with homologous genes from published stonefly species. tRNA genes were identified using the ARWEN online service and checked manually (Laslett and Canbäck 2008). Each protein-coding gene was aligned individually based on codon-based multiple alignments using the MAFFT algorithm within the TranslatorX online platform (Abascal et al. 2010). Before back-translating to nucleotides, poorly aligned sites were removed from the protein alignment using Gblocks in the TranslatorX with default settings. Nucleotide substitution rates, base composition, and codon usage were analyzed with MEGA 6.0 (Tamura et al. 2013). The GC and AT skews were calculated using the formulae: AT skew= (A−T)/ (A+T) and GC skew= (G−C)/ (G+C) (Perna and Kocher 1995).

We calculated the value of Ka (the rates of non-synonymous substitutions), Ks (the rates of synonymous substitutions) using DnaSP 5.1.0 (Librado and Rozas 2009). We also identified tandem repeats in the A+T-rich region using the Tandem Repeats Finder server (Benson 1999), and predicted the stem-loop structure using the mfold web server (Zuker 2003).

### Phylogenetic analysis

The phylogenetic trees among three families of the superfamily Nemouroidea were reconstructed using DNA data from nine published and two newly sequenced mitogenomes. Two stonefly species, *Pteronarcysprinceps* and *Styloperlaspinicercia*, were used as outgroups (Table 1). We assembled the following datasets for phylogenetic analyses: 1) the “PCGs matrix” (total of 11,046 bp), inclusive of 13 PCGs; 2) the “PCG12 matrix” (total of 7,364 bp) which contains the first and second codon positions of protein-coding genes; 3) the “PCGR matrix” (total of 13,056 bp) which contains all three codon positions of protein-coding genes and two ribosomal RNA genes; 4) the “PCG12R matrix” (9374 bp) which contains the first and second codon of PCGs and two ribosomal RNA genes. The BI and ML analyses were carried out on the CIPRES Science Gateway (Miller et al. 2010). ML analyses were performed with RaxML-HPC2 on XSEDE 8.1.10 (Stamatakis 2006) using the GTRGAMMAI model, and node confidence was assessed with 1,000 bootstrap replicates. Bayesian analyses were carried out using MrBayes 3.2.6 on XSEDE (Ronquist et al. 2012). For BI analyses, GTR+I+G was the best-fit model for the nucleotide sequence alignments, using jModelTest 0.1.1 based on Akaike’s information criterion (AIC) (Posada 2008). We conducted with two simultaneous runs for 10 million generations. Each set was sampled every 1,000 generations with a burn-in rate of 25%. Stationarity was examined by Tracer v.1.6 (http://tree.bio.ed.ac.uk/software/tracer/), and was considered to be reached when the ESS (estimated sample size) value was above 200.

**Table 1.** General information of the taxa used in this study.

Family	Species	Number (bp)	Accession number
Nemouridae	* Nemoura nankinensis *	16,602	KY940360
	* Mesonemoura tritaenia *	15,778	MH085451
	* Mesonemoura metafiligera *	15,739	MH085450
Capniidae	* Apteroperla tikumana *	15,564	NC_027698
	* Capnia zijinshana *	16,310	KX094942
	* Mesocapnia arizonensis *	14,921	KP642637†
	* Mesocapnia daxingana *	15,524	KY568983†
Taeniopterygidae	* Doddsia occidentalis *	16,020	MG589787
	* Taeniopteryx ugola *	15,353	MG589786
Styloperlidae (Outgroup)	* Styloperla spinicercia *	16,219	KX845569
Pteronarcyidae (Outgroup)	* Pteronarcys princeps *	16,004	NC_006133

† Incomplete genome sequence.

## Results and discussion

### Genome features

**Figure 1.**
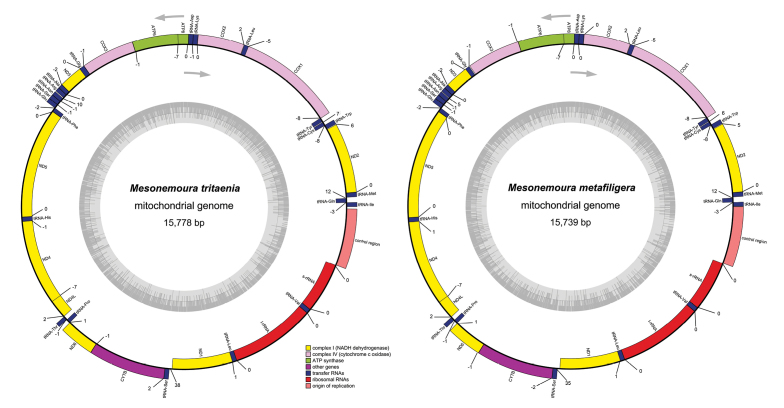
Map of the mitogenomes of *M.metafiligera* and *M.tritaenia*. Genes shown on the inside of the map are transcribed in a clockwise direction, whereas those on the outside of the map are transcribed counterclockwise. Different gene types are shown as filled boxes in different colors. Numbers show the sizes of intergenic spacers (positive values) and overlapping region between genes (negative values).

In the present study, two complete mitogenomes of *M.metafiligera* and *M.tritaenia* were sequenced and deposited in GenBank of NCBI under accession numbers MH085450–MH085451 (see Table 1 and Fig. 1). The *M.metafiligera* mitogenome was a 15,739 bp circular DNA molecule, which was smaller than *M.tritaenia* (15,778 bp) due to differences in the size of the A+T-rich region (Tables 1 and 2). The size of completely sequenced mitogenomes were medium-sized when compared with the mitogenomes of other stoneflies, which ranged from 16,602 bp (*N.nankinensis*) (Chen and Du 2017a) to 15,048 bp (*Isoperlabilineata*) (Chen et al. 2018). All genes identified in the two mitogenomes are typical animal mitochondrial genes with normal gene sizes. Mitochondrial gene order was the same as all previously published stonefly mitogenomes, as well as the ancestral gene order of insects, as exemplified by *Drosophilayakuba* (Clary and Wolstenholme 1985). When compared with the mitogenome of *N.nankinensis* (Chen and Du 2017a), the length variation was limited in the PCGs, tRNA, and rRNA genes, but very different in the A+T-rich region.

In the mitogenome of *M.metafiligera*, 50 overlapping nucleotides were located in 15 pairs of neighboring genes, while in the mitogenome of *M.tritaenia*, there were 51 overlapping nucleotides in 16 gene boundaries. These overlapping nucleotides varied in length from 1 to 8 bp (Table 2). When compared with the mitogenome of *N.nankinensis*, three conserved regions were found in overlapping regions of genes of each sequenced nemourid species and were also observed in some other stoneflies: AAGCCTTA (tRNA^Trp^-tRNA^Cys^), ATGATTA (ATP8–ATP6) and ATGTTAA (ND4–ND4L) (James and Andrew 2006; Qian et al. 2014; Wu et al. 2014; Elbrecht et al. 2015; Huang et al. 2015; Wang et al. 2017a, 2017b, 2018a). The ATP8/ATP6 and the ND4/ND4L gene pairs overlap are also found in many insect mitogenomes and thought to be translated as a bicistron (Stewart and Beckenbach 2005). The longest intergenic spacer we found located between tRNA^Ser(UCN)^ and ND1 in two nemourid species, ranging from 35 bp in *M.metafiligera* to 38 bp in *M.tritaenia* (Table 2). In *Drosophilamelanogaster*, two conserved non-coding intergenic regions (tRNA^Glu^-tRNA^Phe^, and tRNA^Ser(UCN)^–ND1) have been shown to be binding sites for a bidirectional transcription termination factor (DmTTF) (Beckenbach 2012). For the first binding site of tRNA^Glu^-tRNA^Phe^, this region was absent in two nemourid mitogenomes (Table 2). Studies show that this region is absent or incomplete in other plecopteran species as well as in other insect orders (Beckenbach 2012). However, the second binding site of tRNA^Ser(UCN)^–ND1 is found in two nemourid species. This region is more widely conserved and similar non-coding sequences are present at this site in other insect orders (Beckenbach 2012).

**Table 2.** Mitochondrial genome structures of *Mesonemouratritaenia* and *Mesonemourametafiligera*.

Gene	Direction	* Mesonemoura tritaenia *				* Mesonemoura metafiligera *			
		Location (bp)	Size (bp)	Anti– or Start/Stop Codons	IGN	Location (bp)	Size (bp)	Anti– or Start/Stop Codons	IGN
tRNA^Ile^	F	1–67	67	GAT		1–66	66	GAT	
tRNA^Gln^	R	65–133	69	TTG	–3	64–132	69	TTG	–3
tRNA^Met^	F	146–212	67	CAT	12	145–211	67	CAT	12
ND2	F	213–1247	1,035	ATG/TAG	0	212–1,246	1,035	ATG\TAG	0
tRNA^Trp^	F	1,254–1,322	69	TCA	6	1,252–1,320	69	TCA	5
tRNA^Cys^	R	1,315–1,377	63	GCA	–8	1,313–1,375	63	GCA	–8
tRNA^Tyr^	R	1,385–1,450	66	GTA	7	1,382–1,448	67	GTA	6
COI	F	1,443–2,987	1,545	ATT/TAA	–8	1,441–2,985	1,545	ATT\TAA	–8
tRNA^Leu(UUR)^	F	2,983–3,049	67	TAA	–5	2,981–3,047	67	TAA	–5
COII	F	3,052–3,739	688	ATG/T–	2	3,050–3,737	688	ATG\T–	2
tRNA^Lys^	F	3740–3810	71	CTT	0	3,738–3,808	71	CTT	0
tRNA^Asp^	F	3,810–3,877	68	GTC	–1	3,809–3,876	68	GTC	0
ATP8	F	3,878–4,036	159	ATT/TAA	0	3877–4035	159	ATT\TAA	0
ATP6	F	4,030–4,707	678	ATG/TAA	–7	4,029–4,706	678	ATG\TAA	–7
COIII	F	4,707–5,495	789	ATG/TAA	–1	4,706–5,494	789	ATG\TAA	–1
tRNA^Gly^	F	5,495–5,560	66	TCC	–1	5,494–5,559	66	TCC	–1
ND3	F	5,561–5,914	354	ATT/TAG	0	5,560–5,913	354	ATT\TAG	0
tRNA^Ala^	F	5,913–5,976	64	TGC	–2	5,912–5,975	64	TGC	–2
tRNA^Arg^	F	5,977–6,041	65	TCG	0	5,976–6,040	65	TCG	0
tRNA^Asn^	F	6,052–6,116	65	GTT	10	6,046–6,110	65	GTT	5
tRNA^Ser(AGN)^	F	6,116–6,184	69	GCT	–1	6,110–6,178	69	GCT	–1
tRNA^Glu^	F	6184–6249	66	TTC	–1	6,178–6,244	67	TTC	–1
tRNA^Phe^	R	6,248–6,312	65	GAA	–2	6,243–6,307	66	GAA	–2
ND5	R	6,313–8,047	1,735	ATG/T–	0	6,308–8,042	1,735	GTG\T–	0
tRNA^His^	R	8,048–8,113	66	GTG	0	8,043–8,109	67	GTG	0
ND4	R	8,115–9,455	1,341	ATG/TAA	1	8,111–9,451	1,341	ATG\TAA	1
ND4L	R	9,449–9,745	297	ATG/TAA	–7	9,445–9,741	297	ATG\TAA	–7
tRNA^Thr^	F	9,748–9,813	66	TGT	2	9,744–9,809	66	TGT	2
tRNA^Pro^	R	9,813–9,877	65	TGG	–1	9,809–9,873	64	TGG	–1
ND6	F	9,879–10,403	525	ATT/TAA	1	9875–10399	525	ATT\TAA	1
CytB	F	10,403–11,539	1,137	ATG/TAG	–1	10,399–11,535	1,137	ATG\TAG	–1
tRNA^Ser(UCN)^	F	11,538–11,607	70	TGA	–2	11,534–11,603	70	TGA	–2
ND1	R	11,646–12,596	951	TTG/TAA	38	11,639–12,589	951	TTG\TAA	35
tRNA^Leu(CUN)^	R	12,598–12,663	66	TAG	1	12,591–12,656	66	TAG	1
lrRNA	R	12,664–13,992	1,329		0	12,657–13,983	1,327		0
tRNA^Val^	R	13,993–14,063	71	TAC	0	13,984–14,054	71	TAC	0
srRNA	R	14,064–14,857	794		0	14,055–14,846	792		0
A+T-rich region		14,858–15,778	921		0	14,847–15,739	893		0

IGN: Intergenic nucleotides.

Similarly to what observed in other insects, the nucleotide composition of two nemourid mitogenomes was clearly biased towards a higher content of A/T nucleotides. The base composition bias of the *M.metafiligera* mitogenome is 69.1% A + T content, made up of 66.9% in the PCGs, 71.3% in the tRNAs, 72.6% in the rRNAs and 84.1% in the A + T rich region. While the base composition bias of the *M.tritaenia* mitogenome is 68.6% A + T content, made up of 66.4% in the PCGs, 71.3% in the tRNAs, 72.0% in the rRNAs and 82.1% in the A + T rich region (Table 3). This is identical to the base composition biases reported in other stoneflies, which ranges from 62.5% (*Dinocrascephalotes* Curtis, 1827) to 71.5% (*Pteronarcysprinceps* Banks, 1907) (James and Andrew 2006; Elbrecht et al. 2015). After comparing the nucleotide compositions of four major partitions (PCGs, tRNAs, rRNAs, and the A+T-rich region), we found that all four partitions were consistently biased towards A and T. Among the four partitions, the lowest A + T content was found in PCGs, whereas the highest in the A+T-rich region of the two nemourid specimens. The strand bias also can be measured as AT– and GC-skews (Perna and Kocher 1995). Both two nemourid species had positive AT-skew and negative GC-skew values for the entire mitogenome (Table 3), showing a biased use for the A and C nucleotides, which was common in most other insects (Wei et al. 2010).

**Table 3.** The nucleotide composition of *Mesonemouratritaenia* and *Mesonemourametafiligera* mitogenome.

Genes or regions	Size	Nucleotides composition (%)				A+T (%)	AT Skew	GC Skew
		T	C	A	G			
* Mesonemoura tritaenia *								
Complete mitogenome	15,778	32.5	19.0	36.0	12.5	68.6	0.051	–0.208
PCGs	11,199	38.8	17.3	27.6	16.3	66.4	–0.169	–0.029
tRNA genes	1,471	35.9	12.6	35.4	16.1	71.3	–0.007	0.123
rRNA genes	2,123	39.6	9.8	32.4	18.2	72.0	–0.100	0.300
*lrRNA*	1,329	40.6	8.7	32.7	18.1	73.3	–0.109	0.352
*srRNA*	794	37.9	11.7	32.0	18.4	69.9	–0.085	0.222
A+T-rich region	921	39.5	11.8	42.6	6.1	82.1	0.037	–0.321
* Mesonemoura metafiligera *								
Complete mitogenome	15,739	33.0	18.6	36.2	12.3	69.1	0.046	–0.206
PCGs	11,199	39.9	16.5	27.0	16.6	66.9	–0.194	0.000
tRNA genes	1,473	36.0	12.8	35.3	16.0	71.3	–0.010	0.111
rRNA genes	2,119	39.5	9.5	33.1	17.8	72.6	–0.089	0.303
*lrRNA*	1,327	40.5	8.3	33.6	17.6	74.2	–0.093	0.359
*srRNA*	792	37.9	11.6	32.2	18.3	70.1	–0.081	0.224
A+T-rich region	893	39.6	10.4	44.5	5.5	84.1	0.057	–0.310

### Protein-coding genes

The total length of all PCGs of both mitogenomes of *M.metafiligera and M.tritaenia* were 11,199 bp (Table 3). Most PCGs of the two mitogenomes initiated with the typical start codon ATN (Met/Ile), however TTG was proposed as the start codon for ND1 in two species (Table 2). Gene ND1 has been found to employ TTG as a start codon in some insects, thus minimizing intergenic spacing and avoiding overlap with adjacent genes (Bae et al. 2004; Sheffield et al. 2008; Wei et al. 2010). The ND5 gene of *M.metafiligera* has the unusual start codon GTG, as previously reported in other stoneflies (James and Andrew 2006; Elbrecht et al. 2015; Huang et al. 2015; Sproul et al. 2015; Chen et al. 2016; Elbrecht and Leese 2016; Chen and Du 2017b; Wang et al. 2017a, 2017b).

Similar to most other stoneflies, the most commonly used stop codon in two nemourid specimens was TAA, which was found in eight of the 13 PCGs (ATP6, ATP8, COI, COIII, ND1, ND4, ND4L and ND6) for both two nemourid mitogenomes. The stop codon TAG was used in ND2, ND3 and CytB. In *M.metafiligera and M.tritaenia*, both COII and ND5 terminate with incomplete stop codon T (Table 2). The phenomenon of incomplete stop codons is common in insect mitogenomes and it is likely that these truncated stop codons are completed by posttranscriptional polyadenylation (Ojala et al. 1981). Overall, the start and stop codons were similar in two nemourid specimens, and showed little differences between them in some PCGs (Table 2).

The genome-wide bias toward A+T content was also reflected in the codon usage by the PCGs. The relative synonymous codon usage (RSCU) showed high base compositional biases for AT in the mitogenomes of *M.tritaenia* and *M.metafiligera* (Suppl. material 1, Figure S1). The codons ending with A or U were preferred in both the four-fold and two-fold degenerate codons. However, in *M.metafiligera* mitogenomes, the RSCU value of GGG (Gly) was higher than GGT (Gly) (Suppl. material 1, Figure S1). This may be caused by the anticodon tRNA^Gly^-TCC, which contain swinging nucleotides and can decode GGG (Mao and Dowton 2014).

In order to analyze the evolutionary patterns of PCGs in the three nemourid mitogenomes, we compared the ratio of Ka/Ks for each PCGs (Suppl. material 2, Figure S2). The observed average Ka/Ks ratios were consistently lower than 0.3, increasing from 0.025 for ATP6 to 0.202 for ND6 (Suppl. material 2, Figure S2). The uniformly low values of Ka/Ks ratios for COI, COII, COIII and CytB indicate strong evolution constraints in cytochrome c oxidase (Schmidt et al. 2001; Zsurka et al. 2010). The Ka/Ks values for all PCGs were below 0.3, indicating that these genes are evolving under purifying selection. Therefore, all mitochondrial PCGs could be used to investigate phylogenetic relationships within nemourid.

### Transfer and ribosomal RNAs

**Figure 2.**
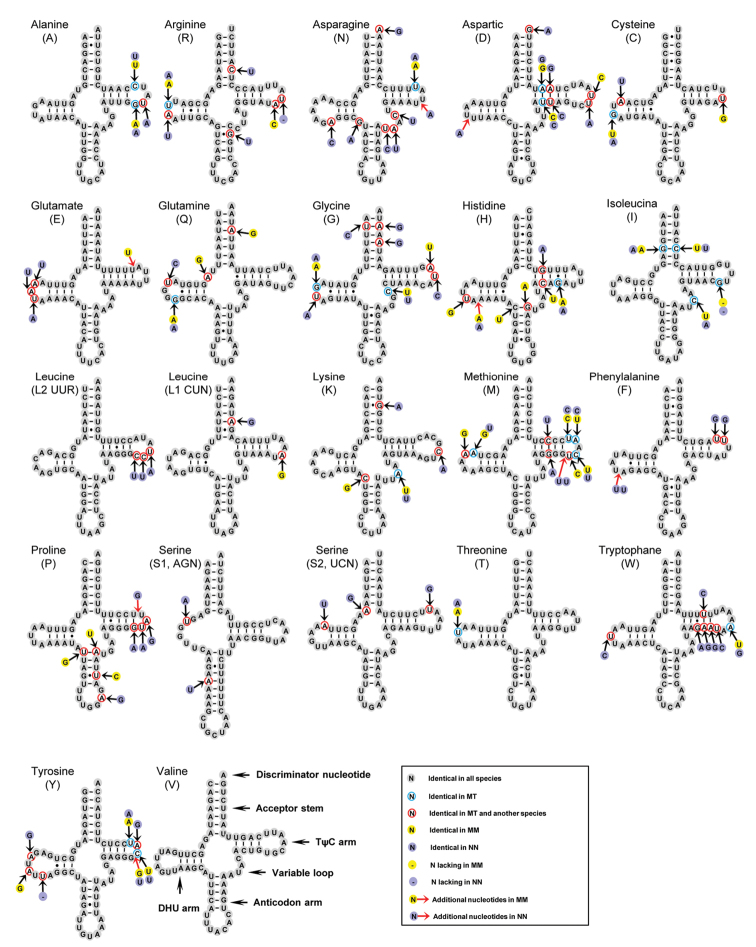
Secondary structure of tRNA families in nemourid mitogenomes. The nucleotide substitution pattern for each tRNA family is modeled using as reference the structure determined for *M.tritaenia*. Red arrows correspond to insertions. Inferred Watson-Crick bonds are illustrated by lines, whereas GU bonds are illustrated by dots.

The sizes of 22 tRNA genes of *M.metafiligera* and *M.tritaenia* range from 63 bp to 71 bp, comprising 9.36% (1,473 bp) and 9.32% (1,471 bp) of the total mitogenomes, respectively (Table 2 and 3). By compared with *N.nankinensis* mitogenome, all 22 tRNA genes in the three nemourid species were identified and the secondary structures were shown in Fig. 2. Most tRNA genes could be folded into a classic clover-leaf secondary structure except for tRNA^Ser(AGN)^ due to a lack of the dihydrouridine (DHU) arm (Fig. 2). The loss of the DHU arm in tRNA^Ser(AGN)^ was a typical feature in metazoan mitogenomes (Negrisolo et al. 2011). However, we found that tRNA^Ser(AGN)^ in the three nemourid mitogenomes possessed an unusual anticodon stem (9 pairs of nucleotides) with an extended nucleotide (Fig. 2). This is an unusual phenomenon, but has also been observed in the DHU arm in other stoneflies (Chen and Du 2017b; Wang et al. 2017a; Wang et al. 2018a, 2018b).

According to the secondary structures and sequence alignment, the most conserved tRNAs in nemourid mitogenomes were tRNA^Val^, tRNA^Thr^, tRNA^Ser(AGN)^ and tRNA^Leu(CUN)^ with no more than two nucleotides substitution in each gene (Fig. 2). Nucleotide insertion-deletion polymorphisms were mainly restricted to TΨC and DHU loops. As expected, tRNAs of *M.tritaenia* and *M.metafiligera* showed high level of sequence and structural identity, with five identical tRNAs (tRNA^Val^, tRNA^Ser(UCN)^, tRNA^Ser(AGN)^, tRNA^Leu(UUR)^ and tRNA^Phe^) (Fig. 2). The eighteen remaining tRNAs only had four insertion-deletion positions in total (one insertion position in the DHU loop of tRNA^His^, each with one insertion position in the TΨC loop of tRNA^Glu^ and tRNA^Tyr^, and one deletion position in the TΨC loop of tRNA^Ile^), and the nucleotide substitutions of individual gene were mostly restricted to 1–3 nucleotide sites, with the exception of tRNA^His^ and tRNA^Met^ with 5 and 6 sites respectively (Fig. 2).

The large subunit ribosome gene (lrRNA) was located between tRNA^Leu(CUN)^ and tRNA^Val^, while the small subunit ribosome gene (srRNA) was flanked by tRNA^Val^ and the A+T-rich region (Fig. 1). Two ribosome genes (srRNA and lrRNA) were identified at 794 and 1,329 bp in *M.tritaenia* and at 792 and 1,327 bp in *M.metafiligera* (Table 2). Our result shows that the size differences in both ribosomal subunits are not distinct among different species.

**Figure 3.**
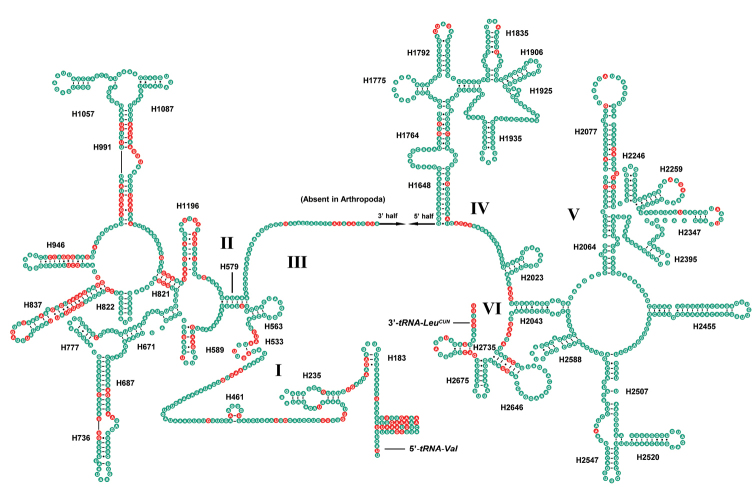
Predicted secondary structure of the *lrRNA* in *M.tritaenia*. Key: red circle, nucleotide conserved in three nemourid mitogenomes; green circle, nucleotide not conserved. Roman numerals represent the conserved domain structures. Dashes (−) indicate Watson-Crick base pairing, and dots (·) indicate G–U base pairing **I–VI** indicate six domains in the secondary structure of lrRNA.

**Figure 4.**
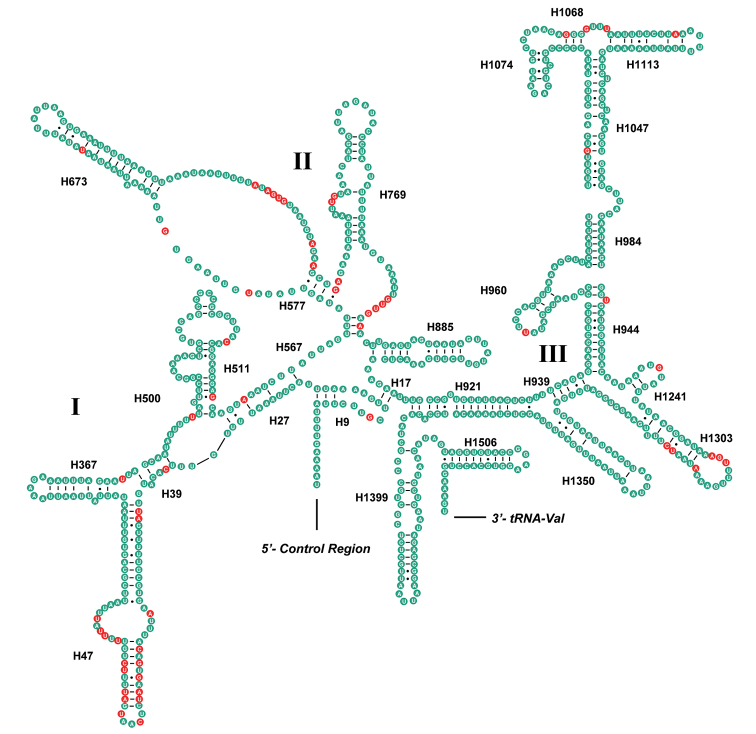
Predicted secondary structure of the *srRNA* in *M.tritaenia*. Key: red circle, nucleotide conserved in three nemourid mitogenomes; green circle, nucleotide not conserved. Roman numerals represent the conserved domain structures. Dashes (−) indicate Watson–Crick base pairing, and dots (·) indicate G–U base pairing **I–VI** indicate six domains in the secondary structure of srRNA.

In this study, the secondary structures of the srRNA and lrRNA of *M.tritaenia* were constructed following the models proposed for other insects, with marked the conserved sites. The secondary structure of lrRNA contained five structural domains (I–II, IV–VI, domain III is absent in arthropods) and 44 helices (Fig. 3). The multiple alignments of nemourid lrRNA genes had 1,334 positions and contained 1,139 conserved positions (85.4%), 190 necleotide substitutions (14.2%) and 5 indels (0.4%), respectively. Nucleotide variability was unevenly distributed among domains and helices, mainly in domains I and II. Several helices (H821, H837, H946, H991, and H1196) in lrRNA, with high variability at the primary sequence level, showed conserved secondary structures. The secondary structure of srRNA contained three domains (I–IV) and 27 helices (Fig. 4). The multiple alignments of nemourid srRNA genes was inclusive of 795 positions and contained 737 conserved positions (92.7%), 56 necleotide substitutions (7.0%) and 2 indels (0.3%), respectively. Nucleotide variability was unevenly distributed among domains and helices, mainly in helices H47 of domain I.

### A+T-rich region

**Figure 5.**
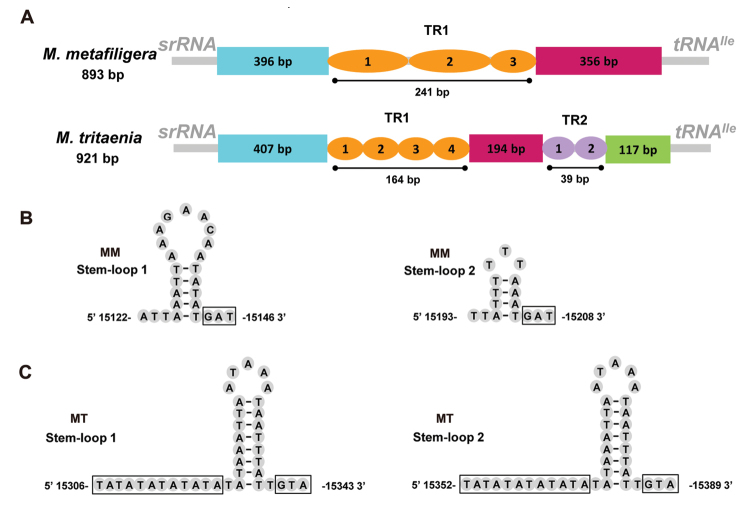
A+T-rich region of two nemourid mitogenomes **A** structure elements found in the A+T-rich region of two nemourid mitogenomes. TR is the abbreviation of tandem repeat units **B** putative stem-loop structures found in the A+T-rich region of *M.metafiligera* (MM indicate *M.metafiligera*) **C** putative stem-loop structures found in the A+T-rich region of *M.tritaenia* (MT indicate *M.tritaenia*).

The A+T-rich regions all locate between srRNA and tRNA^Ile^ in *M.tritaenia* and *M.metafiligera* mitogenome with 921 bp and 893 bp in length, respectively (Table 2). This region is considered to be the control region as it contains both an origin of transcription and replication (Taanman 1999; Cameron 2014). The A+T-rich region is the most variable region in mitogenome due to insertion and deletion events, variation in numbers of tandem repeats, and differences in variable domain length (Zhang et al. 1995; Taanman 1999). In addition, some structural elements are founded in the A+T-rich region, such as poly-T stretch, stem-loop (SL) structures, tandem repeats (TRs) and G+A-rich region, etc. (Zhang and Hewitt 1997). A comparison of molecular features in A+T-rich region between the two newly sequenced mitogenomes was shown in Fig. 5. Some essential elements were observed: (1) a leading sequence adjacent to srRNA; (2) large tandem repeats present as two or more copies; (3) the remainder of the A+T-rich region (Fig. 5A).

Repeated sequences are common in the A+T-rich region for most insects, and length variations are decided to a large degree by the various numbers of repeats (Zhang and Hewitt 1997; Zhang et al. 1995). In the case of *M.tritaenia*, there is a large repeat region (positions: 15,243–15,483) which is 241 bp and contains two tandem repeat units plus a partial copy of the repeat. While in the A+T-rich region of *M.tritaenia* mitogenome, two tandem repeat regions (TR1 and TR2) were founded. TR1 region (positions: 15,265–15,428) is 164 bp and contains three tandem repeat units plus a partial copy of the repeat; In TR2 region (positions: 15,623–15,661), the two tandem repeats units were 21 and 18 bp (not strictly repeats). We also found some SL structures in two newly sequenced species. Two SL structures were predicted in the A+T-rich region of *M.metafiligera* mitogenome: SL–1 (positions: 15,122–15,146) and SL–2 (positions: 15,193–15,208) (Fig. 5B). In the A+T-rich region of *M.tritaenia* mitogenome, we found two long repeated sequences: 5’–TATATATATATATATAAATTAATAAATAATTTATTGTA–3’. Both of them can be folded into a stem-loop structure (SL–1 positions: 15,306–15,343; SL–2 positions: 15,352–15,389) (Fig. 5C). This special structure in the A+T-rich region of *M.tritaenia* mitogenome is quite different with that of other published stoneflies except for *Acroneuriahainana* Wu, 1938, and it may be able to adjust the replication speed of two replicate directions (Huang et al. 2015). The proposed “G(A)_n_T” motif was detected in SL structures of *M.metafiligera* mitogenome, while it was modified as “GTA” in SL structures of *M.tritaenia* mitogenome. In the A+T-rich region of *N.nankinensis* mitogenome, five SL structures with three different motifs (“GTA”, “G(A)_n_T” and “TGA”) were detected (Chen and Du 2017a). The stem-loop structure in the A+T-rich region is identified in many insects and it is thought to be the site of the initiation of minority strand (N-strand) synthesis in *Drosophila* (Clary and Wolstenholme 1987). By contrast, some tRNA-like structures also founded in *N.nankinensis* mitogenome, but these structures were not reported in our newly sequenced species.

## Phylogenetic relationships

**Figure 6.**
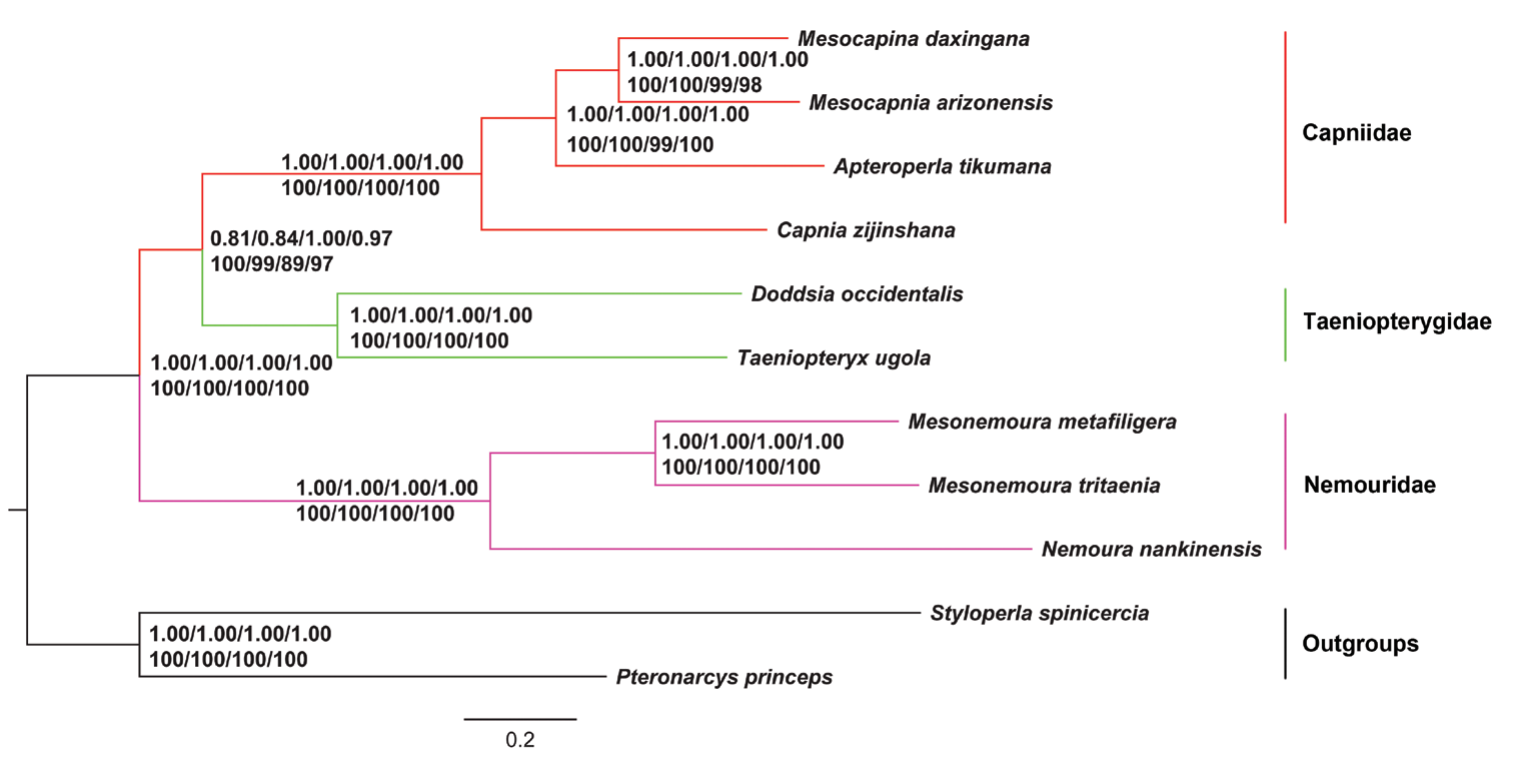
**Phylogenetic tree of the eleven sequenced stoneflies.** Bayesian inference and Maximum Likelihood analysis inferred from PCGs, PCGR, PCG12, and PCG12R supported the same topological structure. Values at nodes are Bayesian posterior probabilities (up) and ML bootstrap values (down) using the PCGs, PCGR, PCG12, and PCG12R datasets. The tree was rooted with two outgroups (*P.princeps* and *S.spinicercia*).

The phylogenetic trees based on Bayesian inference (BI) and maximum likelihood (ML) analyses of PCGs, PCGR, PCG12, and PCG12R datasets were given in Fig. 6. The two methods provided the same tree topology.

In Nemouridae, both ML and BI analyses of four datasets supported the sister-group relationship of Amphinemurinae and the Nemourinae species (Bayesian posterior probabilities (PP) = 1.00/1.00/1.00/1.00; Bootstrap values (BS) = 100/100/100/100), as previous analyses based on the morphological data had indicated (Baumann 1975). The family Capniidae had a closer relationship with Taeniopterygidae (PP = 0.81–1.00; BS = 89–100). However, high intermediate supports were present in both analyses indicating that the phylogenetic position of Capniidae and Taeniopterygidae might be unstable. Meanwhile, the family Nemouridae was placed as sister to Capniidae and Taeniopterygidae (PP = 1.00/1.00/1.00/1.00; BS = 100/100/100/100). Our results of the three families are congruent with our previous results generated by ML analysis of two datasets (PCGR and PCG12) and BI analysis of PCG12 (Wang et al. 2019), and this relationship is also well supported by some studies based on mitogenomes (Chen and Du 2017a) and transcriptome data (Davis 2013), but this placement disagrees somewhat with previous morphological hypotheses. Illies (1965) considered that the Taeniopterygidae was the sister-group to a clade Nemouridae + (Capniidae + Leuctridae). In Zwick’s study, Taeniopterygidae was the sister-group to a clade (Capniidae + Leuctridae) + (Nemouridae + Notonemouridae) (Zwick 2000). In addition, our results are more incongruent with some molecular analysis. For example, Thomas et al. (2000) placed the Nemouridae and the rest of the Nemouroidea as a relatively derived clade, while Terry and Whiting (2003) supported Nemouridae as a sister taxon to Capniidae and then clustered with Taeniopterygidae. For this phenomenon, we believe that more comprehensive mitogenomes of Nemouroidea will solve this problem.

## Conclusions

This study characterized two complete mitogenomes of the subfamily Amphinemurinae. The study provided the following conclusions: (1) Mitochondrial gene order of two Amphinemurinae species was the same as all previously published stonefly mitogenomes, as well as the ancestral pattern of insects, as exemplified by *D.yakuba*. (2) The evolutionary patterns of PCGs were observed in the three nemourid mitogenomes, which may indicate that these genes are evolving under purifying selection. (3) Novel structure characteristics were observed in the mitogenomes. In the two Amphinemurinae mitogenomes, stem-loop structures and tandem repeat sequences were detected. (4) Phylogenetic analysis supported that Amphinemurinae and Nemourinae were sister-group and the family Nemouridae was placed as sister to Capniidae and Taeniopterygidae. This study increases the understanding of stonefly phylogeny.

## Supplementary materials

Supplementary material 1 Figure S1. Relative synonymous codon usage (RSCU) of each amino acid in the mitogenomes of *M.tritaenia* and *M.metafiligera* Data type: phylogenetic data Explanation note: Codon families were provided on the x-axis. Stop codon is not given. This dataset is made available under the Open Database License (http://opendatacommons.org/licenses/odbl/1.0/). The Open Database License (ODbL) is a license agreement intended to allow users to freely share, modify, and use this Dataset while maintaining this same freedom for others, provided that the original source and author(s) are credited.Jin-Jun Cao, Ying Wang, Yao-Rui Huang, Wei-Hai Li

Supplementary material 2 Figure S2. Ka/Ks ratios of PCGs in the three nemourid mitogenomes Data type: molecular data Explanation note: The blue columns indicate the average Ka/Ks for each gene. This dataset is made available under the Open Database License (http://opendatacommons.org/licenses/odbl/1.0/). The Open Database License (ODbL) is a license agreement intended to allow users to freely share, modify, and use this Dataset while maintaining this same freedom for others, provided that the original source and author(s) are credited.Jin-Jun Cao, Ying Wang, Yao-Rui Huang, Wei-Hai Li
