# Supplementary figures and images for: Mitochondrial genomes of the stoneflies Mesonemourametafiligera and Mesonemouratritaenia (Plecoptera, Nemouridae), with a phylogenetic analysis of Nemouroidea
Source: Zookeys. 2019 Apr 4;835:43–63. doi: 10.3897/zookeys.835.32470 (PMC6477857; doi:10.3897/zookeys.835.32470)

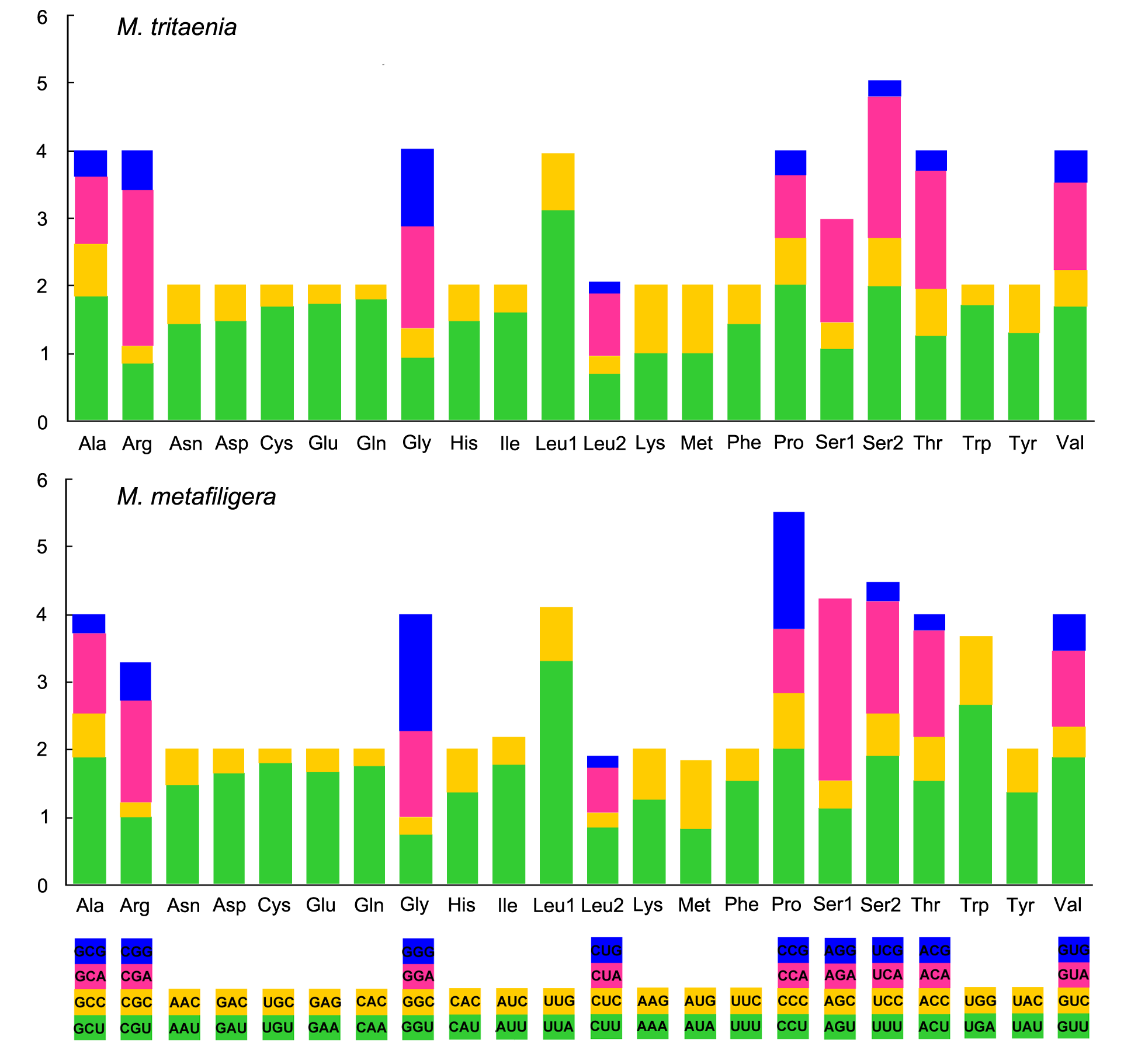

Supplement: Supplementary material 1 [file zookeys-835-043-s001.tif]

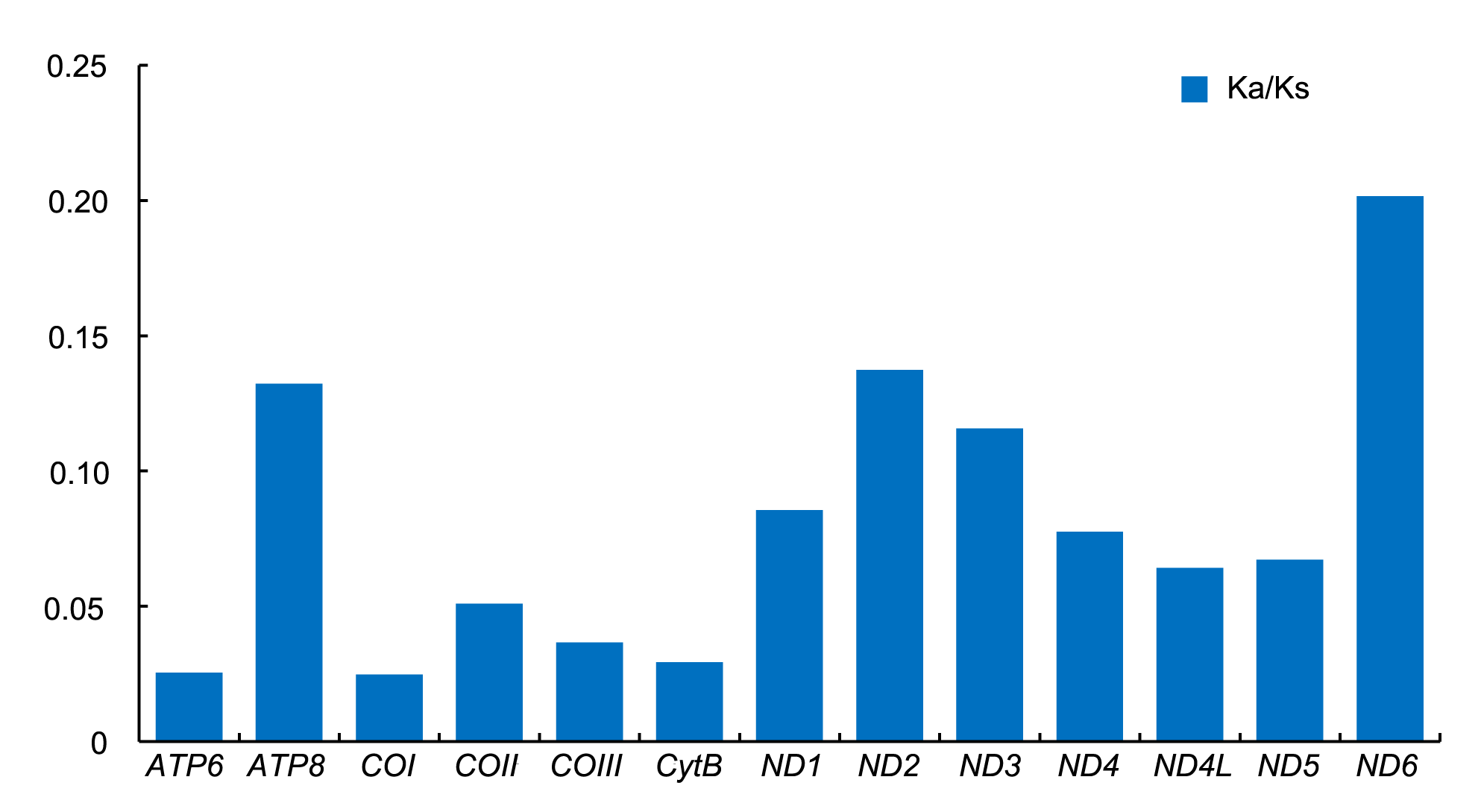

Supplement: Supplementary material 2 [file zookeys-835-043-s002.tif]
